# Supplementary material for: Soda lignin as a sustainable photosensitive component for conventional and controlled radical photopolymerization
Source: Commun Chem. 2025 Jul 5;8:199. doi: 10.1038/s42004-025-01593-0 (PMC12228747; doi:10.1038/s42004-025-01593-0)
Supplement: Supplementary file 2 — Supplementary Information [file 42004_2025_1593_MOESM2_ESM.pdf]

Table of Supplementary Contents

1. Figures and Complementary Explanations....2
1.1 Complementary spectroscopic, photophysical, photoelectrical, and electrochemical experiments....2
1.2 Cyclic voltametric curves of $CuBr_2$/L and $CuBr_2$/L with AL....3
1.3 Cyclic voltametric curve of CuCl2/L with AL....3
1.4 Fluorescence measurements....4
1.5 Fluorescence decay times....5
1.6 Stern-Volmer Plots obtained after the addition of Metal halides....6
1.7 Molecular weight distribution of polymers obtained with metal-free system and respective chain extension experiments....9
1.8 XPS analysis of lignin treated with Cu^{2+} and Fe^{3+}....10
1.9 NMR spectra....11
1.10 XPS spectra of arylsulfonated lignin (AL-SO_3)....12
2. Photopolymerization with $FeBr_3$....14
Supplementary References....15

1. Figures and Complementary Explanations

1.1 Complementary spectroscopic, photophysical, photoelectrical, and electrochemical experiments

Figure S1 shows the photophysical and photoelectrochemical properties of **AL**. The addition of $CuBr_2$ increased the absorbance of **AL** (Figure S1a) and decreased the fluorescence intensity of **AL** (Figure S1b). Adding of $CuBr_2$ decreased the oxidation potential of **AL**, confirming the electron transport occurred between **AL** and $CuBr_2$ (Figure S1c). These properties lay a foundation for **AL** to be used as a photosensitizer.

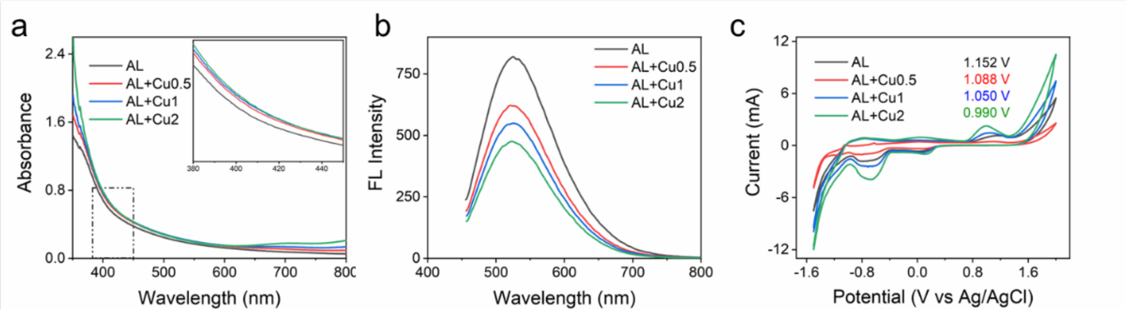

Figure S1. Photophysical and photoelectrochemical properties of **AL**. a) UV-vis absorbance of **AL** and **AL** with different content of $CuBr_2$; b) Fluorescence spectra of **AL** with different content of $CuBr_2$; c) Cyclovoltammetric curves of **AL** with different loading on $CuBr_2$/**L** (Cu0.5 relates a Cu^{2+} content of 25 ppm; Cu1 relates a Cu^{2+}content of 50 ppm; Cu2 relates to a Cu^{2+}content of 100 ppm, the molar ratio of $CuBr_2$ and Ligand here was 0.03:0.135, same with the reaction conditions).

1.2 Cyclic voltametric curves of $CuBr_2$/L and $CuBr_2$/L with AL

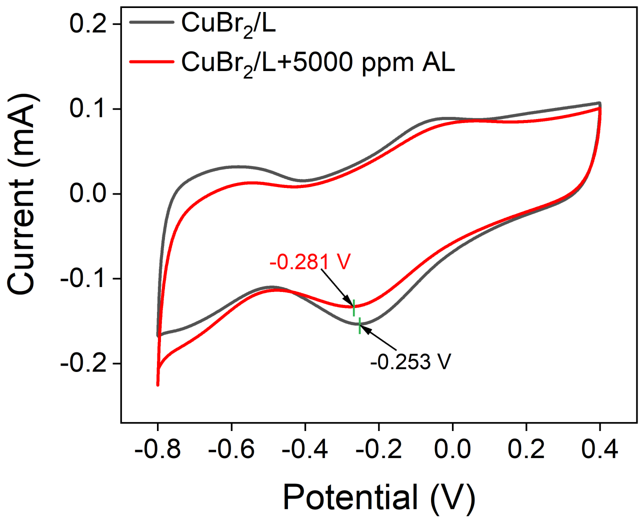

Figure S2. Cyclic voltammetric curves of $CuBr_2$/L and $CuBr_2$/L with 5000 ppm AL.

1.3 Cyclic voltametric curve of CuCl2/L with AL

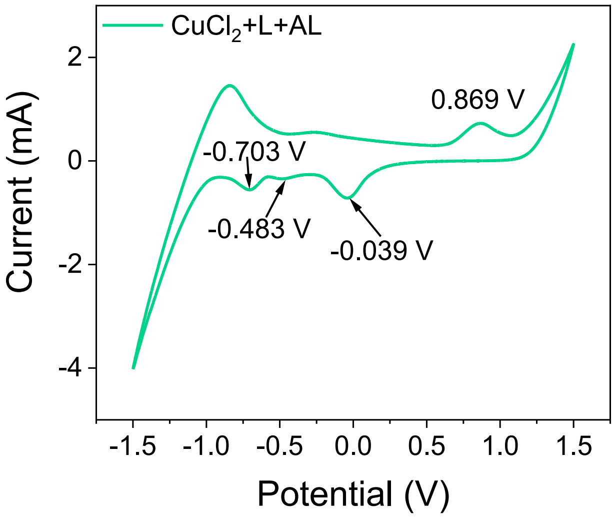

Figure S3. Cyclic voltammetric curve of $CuCl_2$/L with AL.

1.4 Fluorescence measurements

The fluorescence spectra were measured with a PerkinElmer LS 55 Fluorescence Spectrometer. All data were taken in 1×1 cm quartz cuvettes. The fluorescence quenching experiments were evaluated by change of fluorescence intensity changes after adding the quencher to the initial target. Here, the initial fluorescence intensity was taken for **AL** dispersed in DMSO followed by adding the quencher ($CuBr_2$, $FeBr_3$ and **EBPA**) by gradual increase of quencher concentration. This resulted in fluorescence spectra exhibiting different intensities needed for further evaluation of the by the Stern-Volmer plot.

Figure S4 shows the change of fluorescence spectra of **AL** by increasing the content of $CuBr_2$ (Figure S4a), $FeBr_3$ (Figure S4b), and **EBPA** (Figure S4c), respectively. Apparently, all three quenchers decreased the fluorescence intensity of **AL**. The higher the concentration of quenchers, the larger the decrease in fluorescence intensity.

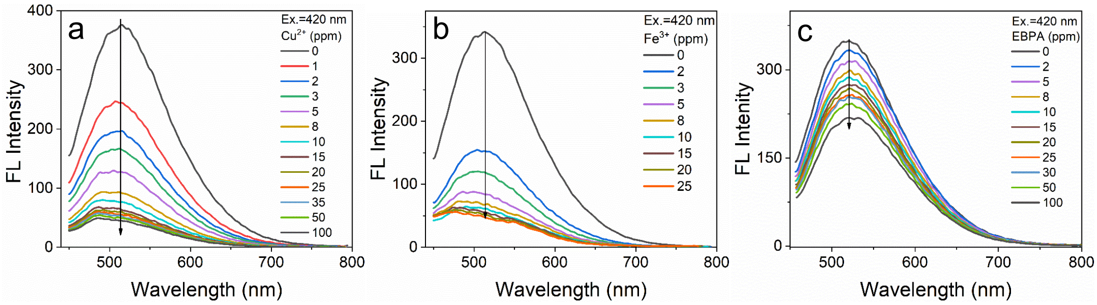

Figure S4. Fluorescence spectra of **AL** quenched with $CuBr_2$, $FeBr_3$, and EBPA. a) **AL** quenched with different content $CuBr_2$; b) **AL** quenched with different content $FeBr_3$; c) **AL** quenched with different content of EBPA. The spectra were obtained under excitation of 420 nm.

1.5 Fluorescence decay times

Figure S5 exhibits the fluorescence decay curves of **AL** and **AL** quenched with $CuBr_2$, $FeBr_3$ and **EBPA,** respectively. Fluorescence lifetimes were obtained by service measurements from Yunpingtai. All three quenchers enabled the decrease of lifetime of **AL**. In the case of **EBPA**, the lifetime decreased most, indicating dynamic quenching. For the other two cases, it was static quenching.

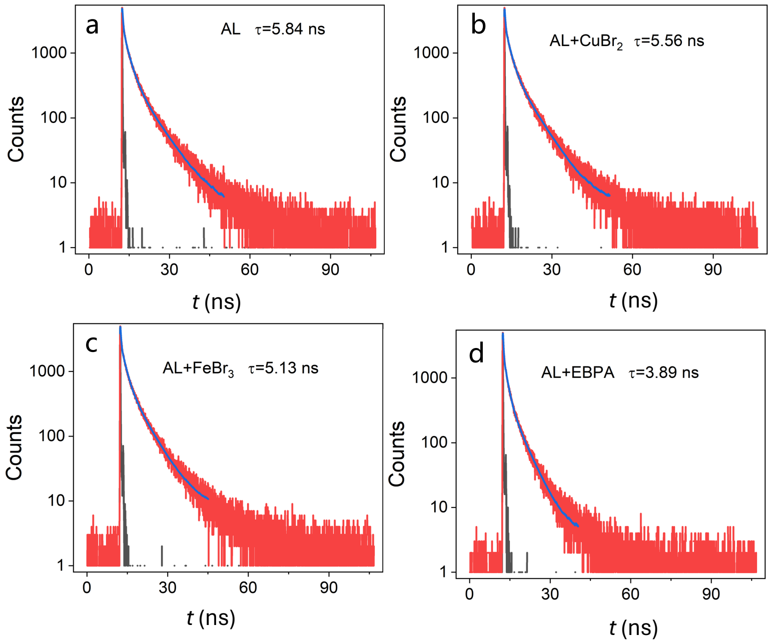

Figure S5. Fluorescence decay curves of **AL** in DMSO (red) and respective instrumental response (black). a) **AL** in DMSO; b) **AL** with $CuBr_2$; c) **AL** with $FeBr_3$; d) **AL** with EBPA. Here, the content of **AL** was 100 ppm and the addition of Cu^{2+}, Fe^{3+} and **EBPA** were 89 mM, 157 mM, and 22 mM, respectively, and relates to the concentration in the Stern-Volmer plots when it leaves the linearity.

1.6 Stern-Volmer Plots obtained after the addition of Metal halides

Such heterogeneous reaction systems additionally require probing events proceeding on a molecular scale to understand better reaction conditions at the rough surface. Quenching of intrinsic fluorescence of **AL** operated as a tool to receive deeper patterns regarding the fractional accessibility (f_a)[1] of the **AL** surface. Stern-Volmer plots (Eq. 2) did not show linearity in the case of the addition of Cu^{2+}, and **EBPA** operating as fluorescence quencher Q, Figures S6a-b. This does not appear as unusual as disclosed for complex biological systems with fluorescence spectroscopy[1]. Here, Eq. 3[1] provides a linear correlation between the respective fluorescence intensities $I_0/(I_0-I)$ and 1/[Q]. This includes f_a, Figures S6c-d, and complementing. Thus, f_a monitors accessibility for fluorescence quenching on the surface probing the surface reactivity by intrinsic fluorescence. Figure S4 shows the respective spectra.

$$I_0/I = 1 + K_{SV}[Q] \qquad \text{(S1)}$$

(K_{SV} = Stern-Volmer constant, [Q] = quencher concentration)

$$\frac{I_0}{I_0-I} = \frac{1}{f_a \times K_{SV}[Q]} + \frac{1}{f_a} \qquad \text{(S2)}$$

The fluorescence decay time (τ_f) only decreased in the case of **EBPA** addition from 5.8 ns to 3.9 ns indicating dynamic quenching. The quenching constant of 1.4×10^{10} $M^{-1}s^{-1}$ remained in the range of diffusion control. On the other hand, the addition of Cu^{2+} and Fe^{3+} resulted only in a small decrease of τ_f to 5.6 ns and 5.1 ns, respectively, see Figure S5. This related to static quenching caused by complexation of Q at the reactive surface. Here, K_{sv} related to the complex building constant to bind $CuBr_2$ and $FeBr_3$ on **AL**. This was 39 M^{-1} and 45 M^{-1}, respectively (Figures 4a-b and S6a-b). Both cationic quenchers indicated larger f_a compared to **EBPA**. FeBr3 was included in first experimental designs because alternative systems showed promising results in sensitized photo-ATRP experiments[2].

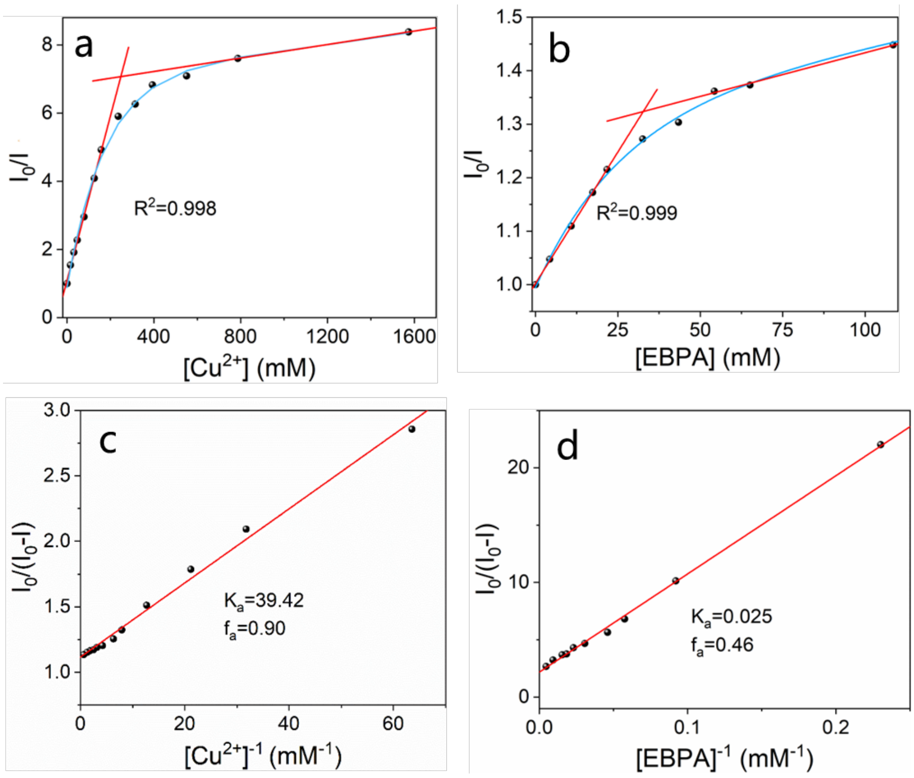

Figure S6. Stern-Volmer plots (Eq. 2) for fluorescence quenching of **AL** after addition of Cu^{2+}, and **EBPA**, as shown in Figure S6a-b, respectively. Eq. 3 successfully discloses the linearity of fluorescence decrease using Cu^{2+} and **EBPA**.

Figure S7a shows the Stern-Volmer plot of **AL** fluorescence quenched by Fe^{3+} based on Eq. 2 in the main manuscript ($I_0/I \sim [Fe^{3+}]$). It did not follow complete linear correlation. Using of Eq. 3 ($I_0/(I_0-I) \sim [Fe^{3+}]^{-1}$), the obtained Stern-Volmer plot showed good linearity (Figure S7b). See the main article for the details of Eq.2 and Eq. 3.

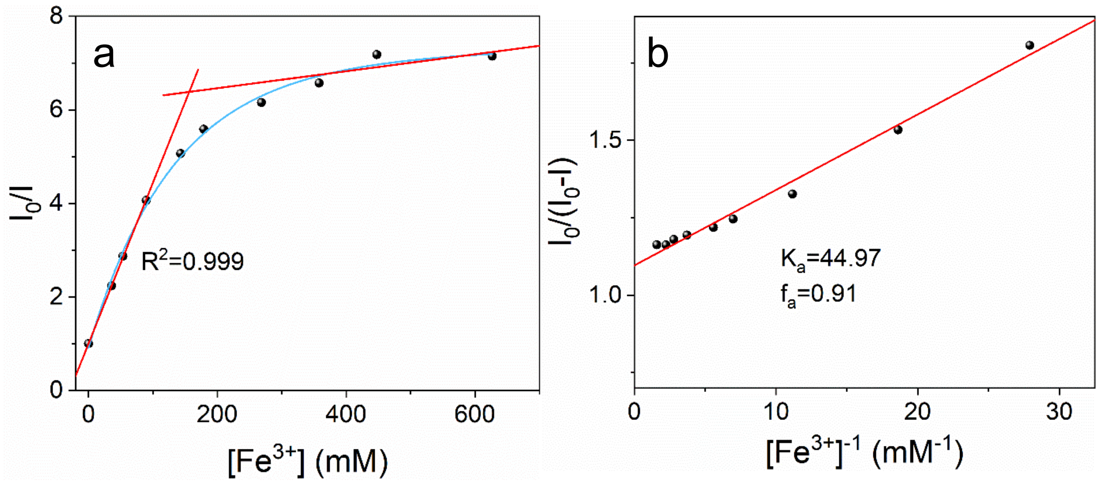

Figure S7. Stern-Volmer plots (Eq. 2) of fluorescence intensity quenching of **AL** document no linearity after adding of Fe^{3+}, Figure S7a. Eq. S2 successfully discloses the linearity of fluorescence changes in Figure S7b using Fe^{3+} as quencher.

1.7 Molecular weight distribution of polymers obtained with metal-free system and respective chain extension experiments

The metal-free photo polymerization procedure was the same as the general procedure, see the Method part in manuscript. The only difference was that $CuBr_2$ and ligand was not added in this procedure. From Figure S8 one can see that an increase of the content of the initiator **EBPA**, the GPC chromatogram showed a bimodal distribution (Figure S8a) where the contribution of lower molecular fraction increased with increased initiator loading. A plot of the respective molecular weight can be seen in Figure S8b exhibiting a wide distribution. In the chain extension experiment with **MMA** in the metal-free system, the shape of the bimodal curve changed in the GPC to one peak with a shoulder and the molecular weight tended to higher values with no significant change of distribution. Presumably, free radical polymerization dominated under these conditions while just a little fraction of polymers was chain extended.

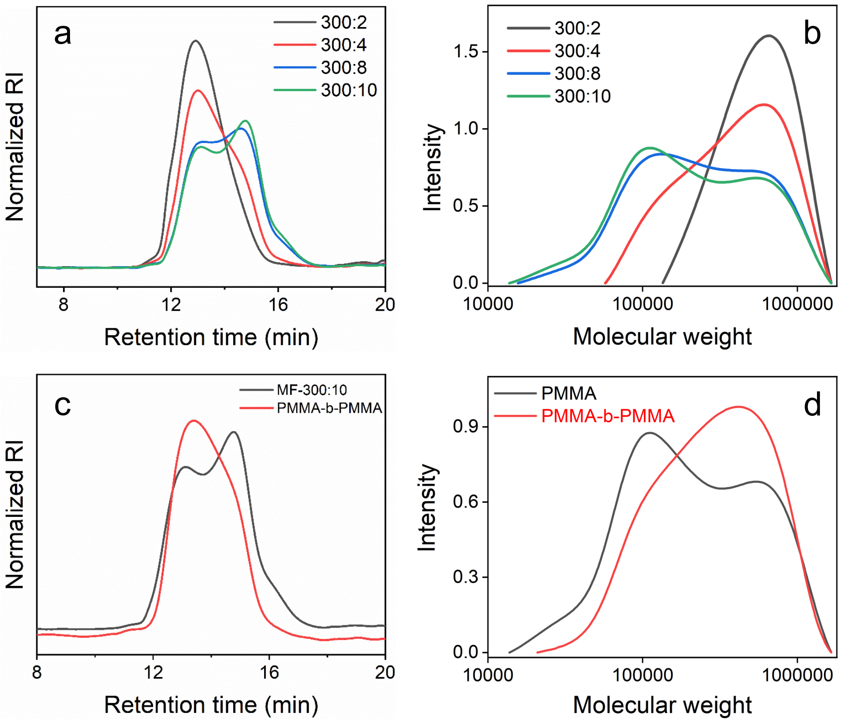

Figure S8. GPC chromatograms of metal free polymerization with different ratio of initiator (a+b) and chain extension with **MMA** (c+d).

1.8 XPS analysis of lignin treated with Cu^{2+} and Fe^{3+}

Figure S9 shows the XPS spectra of **AL** from the Cu-catalytic system and Fe-catalytic system, respectively. The relative content of every element was marked in blue. Apparently, the content of iron (1.98%) was higher than copper (0.18%), proving the higher binding between iron and **AL** than copper. It may be the possible reason why the Fe-catalytic system did not perform than the Cu-catalytic system. Table S1 shows the results obtained for the $FeBr_3$ based system.

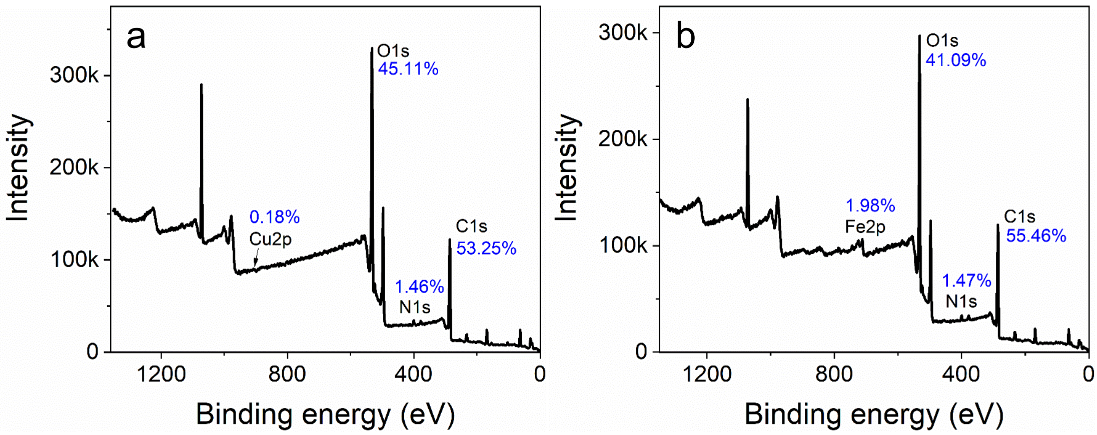

Figure S9. Full scale XPS spectra of AL separated from the Cu-catalytic system after reaction (a) and Fe-catalytic system after reaction. The blue numbers show the relative content of every element in the structure.

1.9 NMR spectra

^{1}H-NMR spectra were taken at an Avance 500 from Bruker. 20 mg sample were dissolved in 0.7 mL $CDCl_3$.

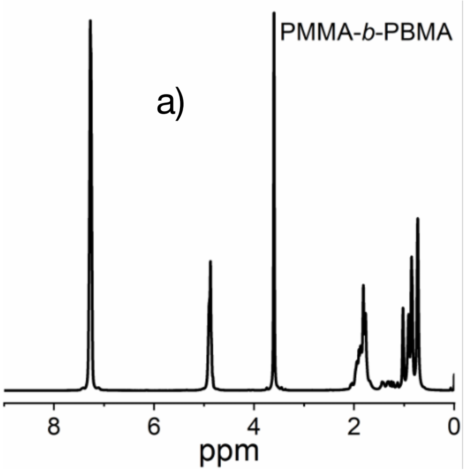

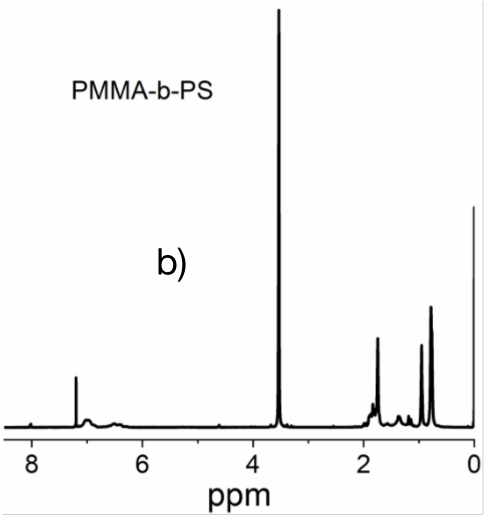

Figure S10. ^{1}H-NMR of the block copolymers **6d** and **6e** in $CDCl_3$.

1.10 XPS spectra of arylsulfonated lignin (AL-SO₃)

The XPS spectra of **AL** and **AL-SO_3** showed that the content of S in **AL-SO_3** (6.64%) was much higher than that in **AL** (1.98%). Accordingly, the content of O decreased from 36.02% to 24.45% after the reaction, confirming the successful preparation of aryl sulfonated lignin. The content of N can be explained by the reaction conditions because TEA was used in the reaction. It was possible for N to remain in the product after the reaction.

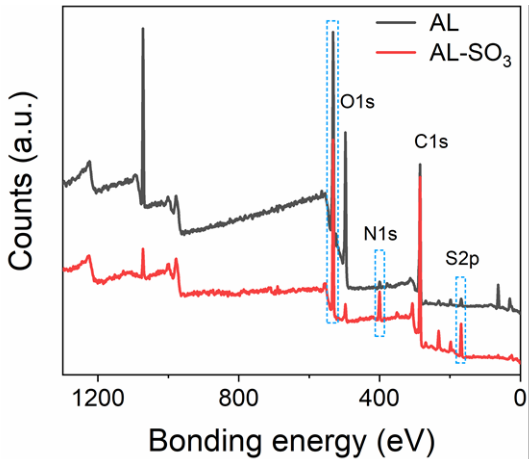

	AL (%)	AL-SO_3 (%)
C	60.16	60.74
N	1.85	8.17
O	36.02	24.45
S	1.98	6.64

Figure S11. Full scale XPS spectra of **AL** and arylsulfonated lignin (**AL-SO_3**) (left) and relative content of every element in the table (right).

2. Photopolymerization with $FeBr_3$

Table S1 shows the experimental conditions and results of Fe-catalyzed photopolymerization using **AL** as the photosensitizer. The experimental procedure was the same as Cu-catalyzed photopolymerization except the catalyst, ligand and the amount of reactant added differed, see supporting information 1.2.1 for the detailed procedure. The higher amount of $FeBr_3$ bound on **AL** results in less available free $FeBr_3$/L catalyst converting the conditions more to those disclosed for metal-free conditions because enough initiator is still available. The iron ions bound on **AL** possibly possess different activation and deactivation constants resulting in unfavorable conditions for the ATRP.

Table S1. Photoinduced Fe-catalyzed ATRP using **AL** in different experimental conditions. Reactions were conducted in 75 vol % DMSO. The irradiation was carried out under blue light LED (420 nm, 35 mW/cm^2). Number average molecular weight (M_n) and dispersity ($Đ$) were obtained by gel permeation chromatography (GPC) in THF using poly(methylmethacrylate) as standard.

No.	**AL** (mg/mL)	[M]:[I]:[$FeBr_3$]:[**L**]	[**L**]	[M]	Time (h)	Conv. (%)	M_n (kDa)	$Đ$
1	1.5	100:1:0.02:0.02	TBABr	**MMA**	24	0.46	69.7	2.99
2	1.5	100:1:0.04:0.04	TBABr	**MMA**	24	5.30	64.3	3.27
3	1.5	100:1:0.08:0.08	TBABr	**MMA**	24	35.79	54.6	3.92
4	1	100:1:0.04:0.04	TBABr	**MMA**	24	51.14	48.5	3.46
5	2	100:1:0.04:0.04	TBABr	**MMA**	24	19.50	47.8	5.28

Supplementary References

1 Lakowicz, J. R. *Principles of Fluorescence Spectroscopy*. (Springer, 2006).

2 Kütahya, C., Meckbach, N., Strehmel, V. & Strehmel, B. Cyanines comprising barbiturate group facilitate NIR - light assisted ATRP under anaerobic and aerobic conditions at two wavelengths using Fe(III) catalyst. *J. Polym. Sci.* **59**, 2023-2035 (2021). https://doi.org:10.1002/pol.20210273
