# Supplementary material for: Soda lignin as a sustainable photosensitive component for conventional and controlled radical photopolymerization
Source: Commun Chem. 2025 Jul 5;8:199. doi: 10.1038/s42004-025-01593-0 (PMC12228747; doi:10.1038/s42004-025-01593-0)
Supplement: Supplementary file 3 — Description of Additional Files [file 42004_2025_1593_MOESM3_ESM.pdf]

Description of Additional Supplementary Files

File name: Supplementary Data 1

Description:

Source data of Figures 1-7 in manuscript.
